# Physicochemical and antioxidative characteristics of Iranian pomegranate (*Punica granatum* L. cv. Rabbab-e-Neyriz) juice and comparison of its antioxidative activity with *Zataria multiflora *Boiss essential oil

**Published:** 2014

**Authors:** Behnaz Bazargani-Gilani, Hossein Tajik, Javad Aliakbarlu

**Affiliations:** *Department of Food Hygiene and Quality Control, Faculty of Veterinary Medicine, Urmia University, Urmia, Iran.*

**Keywords:** Antioxidative activity, Pomegranate juice, Shelf life, Taste and aroma, *Zataria multiflora *Boiss essential oil

## Abstract

Pomegranate juice (PJ) and its products are directly used in foods due to their pleasant taste and palatability as well as preservative effects. In spite of useful effects of essential oils such as *zataria multiflora *Boiss essential oil (ZEO) on prolonging shelf-life of foods, their application is restricted due to their vigorous taste and aroma. In the present study, physicochemical characteristics, chemical compositions and antioxidative activities of two Iranian native plants, PJ (Rabbab-e-Neyriz cultivar) and ZEO were investigated. 2, 2-diphenyl-1-picrylhydrazyl (DPPH) radical scavenging and reducing power tests were used for measuring antioxidant activity. The level of total phenolic of them were also determined. Total soluble solids content, pH value, titratable acidity content and total anthocyanins content of PJ were also measured. Chemical compositions of ZEO were determined using gas-chromatography, mass-spectrometry (GC-MS). The results of antioxidative tests indicated that the ZEO was significantly more potent (*p* < 0.05) than PJ. Also the phenolic content in ZEO (262.52 mg per g) was significantly higher (*p* < 0.05) than PJ (154.90 mg per 100g). Chemical compositions analysis of ZEO indicated that its major components were carvacrol (59.17%), linalool (23.67%), trans-caryophyllene (3.07%) and carvacrol methyl ether (2.44%). In the present study, physicochemical and antioxidative characteristics of Rabbab-e-Neyriz PJ were determined for first time. It was aslo found that ZEO in comparison with PJ had higher antioxidative activity and total phenolic content.

## Introduction

Natural preservatives with high antioxidant activities that prolong the shelf life of food are valuable.^1^ Some alternatives such as modified atmosphere packaging, gamma irradiation, organic acids, ozone treatment, heat, steam or hot water have been found to be effective on shelf life extension in fresh or processed foods.^2^ However, acidification by using organic acids or natural acidic fruit juices is another alternative that is used extensively in food processing to increase the shelf life.^3^ The increasing demand for natural preservatives has resulted in their extended utility.^4^ The various chemical disinfectants are generally undesired by consumers because of their side effects.^4^ Thus, natural sanitizers such as vinegar, lemon juice and pomegranate juice, not only give flavor to foods but they also have the advantage of being a natural preservative.^4^

Pomegranate (*Punica granatum* L.) from the Punicaceae family is an important commercial fruit crop that is extensively cultivated in parts of Asia, North Africa, the Mediterranean and the Middle East.^5^ Iran is one of the most important pomegranate producers and exporters in the world.^6^ The edible parts of pomegranates (called arils) make up approximately 50.00% of the fruit weight and are composed of 76.00 to 85.00% juice and 15.00 to 24.00% seeds.^7^ Recently, the high antioxidant activities of different parts of pomegranate fruit such as juice, peel and seeds have been determined.^7^ The antioxidative activity of pomegranate juice is higher than other fruit juices.^8^ This antioxidant activity has been correlated to the great amount of phenolic compounds, including anthocyanins (3-glucosides and 3,5-diglucosides of delphinidin, cyanidin, and pelargonidin), ellagic acid, punicalin, punicalagin, pedunculagin and different flavanols. The various parts of pomegranate fruits can be consumed fresh or used for the producing of fresh juice, canned beverages, jelly, jam and paste and also for food additive such as flavouring and coloring.^9^ Pomegranate derivatives are the most popular products used to give flavor to several foods such as salads and appetizers, in Turkey.^10^ Rabbab-e-Neyriz is one of the most important cultivars in Iran and we could not find any information regarding the antioxidative activity, physicochemical characteristics and its application with *zataria multiflora* Boiss essential oil in foods.

*Zataria multiflora *Boiss belongs to the family of Lamiaceae, and is an aromatic medicinal plant that grows widely in warm and mountainous parts of Iran, Pakistan and Afghanistan.^11^ The essential oil (EO) of this plant (ZEO) has high quantities of phenolic oxygenated monoterpens and exhibits antioxidant, antibacterial and antifungal activities in *in* *vitro*.^11^ The use of EO for food preservation is often restricted because of high costs, vigorous aroma and also potential toxicity.^12^ An alternative approach to decrease the amounts of essential oils with remaining their benefits in food could be to incorporate them into the formulation of edible coatings.^12^ The aim of this study was to identify the physicochemical and antioxidative characteristics of Iranian pomegranate (*Punica granatum* L. cv. Rabbab-e-Neyriz) juice and comparison of its antioxidative activity with ZEO as native Iranian natural preservative. 

## Materials and Methods

**Preparation of pomegranate juice. **Pomegranate fruit ‘Rabbab-e-Neyriz’ was harvested at the commercial harvest stage from a commercial orchard located in Neyriz, east of Shiraz in the Fars province, Iran. On the same day, harvested fruit were transported by a ventilated car to the Laboratory of Department of Food Hygiene and Quality Control, Faculty of Veterinary Medicine, Urmia University. Fresh pomegranate fruits were washed and cut into four pieces. Separated arils were ground in a mixer for 30 sec and passed through muslin cloth. The freshly prepared juice was used for analyzing.

**Extraction of essential oil and gas chromatography mass spectrometry (GC-MS) analysis.** The plant, *Zataria multiflora *Boiss, was provided by the local groceries and identified at the Institute of Medicinal Plants, Karaj, Iran.^13^ The dried aerial parts were subjected to hydrodistillation for 3 hr using a clevenger-type apparatus. The extracted oil was dried over anhydrous sodium sulfate, followed by filtering and stored in air-tight glass vials covered with aluminum foil at 4 ˚C.^13^ The constituents of EO were identified by a gas chromatograph (GC) (Model 6890N; Hewlett-Packard, Palo Alto, USA) equipped with a column HP-5MS (30 m length × 0.25 mm inner diameter, film thickness 0.25 µm) coupled to a mass spectrometer (MS) (Model 5973N; Hewlett-Packard, Palo Alto, USA). The chromatographic condition was as follows: helium flow rate was 1.5 mL per min; temperature programmed to rise from 50 to 240 ˚C with a gradient of 3 ˚C per min (holding the initial and final temperature for 6 min); followed by a temperature enhancement of 15 ˚C per min up to 300 ˚C (holding for 3 min); injector port temperature and detector temperature were set at 290 ˚C and 250 ˚C, respectively. The individual compounds were identified and confirmed with those of authentic samples and with available library data of the GC/MS system (WILEY 2001 data software; John Wiley and Sons, New York, USA).

**Chemicals and reagents. **Gallic acid, 2,2-diphenyl-1-picrylhydrazyl (DPPH), 2,6,-di-tert-butyl-4-methylphenol (butylated hydroxytoluene, BHT), ferric chloride, and potassium ferricyanide [K_3_Fe(CN)_6_] were obtained from Sigma-Aldrich Chemie (Steinheim, Germany). Analytical grade ethanol, methanol, Folin-Ciocalteu’s phenol reagent, disodium hydrogen phosphate (Na_2_HPO_4_), sodium dihydrogen phosphate (NaH_2_PO_4_), sodium carbonate, anhydrous sodium sulfate, and trichloroacetic acid were purchased from Merck (Darmstadt, Germany).

**DPPH radical scavenging activity. **The ability to scavenge DPPH radical by PJ and ZEO was estimated using the method of Blois with slight modification.^14^ A volume of 50 µL of PJ (1:10) and ZEO (1, 2, 4 and 8 mg mL^-1^) which have been previously dissolved in methanol were mixed with 2 mL of DPPH (24 µg mL^-1^) solution and shaken. The reaction mixture was stored in the dark at room temperature for 60 min and the absorbance was measured at 517 nm by a spectrophotometer (Model Novaspec II; Pharmacia LKB, Uppsala, Sweden). The radical scavenging activity (RSA) was calculated by the following equation:

$\mathrm{RSA}\left(\%\right)=\frac{\left(A_{\mathrm{blank}}-A_{\mathrm{sample}}\right)}{A_{\mathrm{blank}}}\times 100$

where A_blank_ is the absorbance of control reaction (containing all reagents except the test compound), and A_sample_ is the absorbance of the test compound. Butylated hydroxytoluene (1 mg mL^-1^) was used as a positive control. All tests were carried out in triplicate and results were reported as mean ± SD of triplicates.

**Reducing power.** The reducing power of PJ and ZEO were determined according to the method of Oyaizu.^15^ A volume of 1 mL of PJ (1:10) and ZEO (2, 4 and 8 mg mL^-1^) diluted with methanol was mixed with 2.5 mL of sodium phosphate buffer (0.2 M, pH 6.6) and 2.5 mL of potassium ferricyanide (1.0%). After incubation at 50 ˚C for 20 min, 2.5 mL of trichloroacetic acid (10.0%) was added to the mixture to stop the reaction, followed by centrifugation at 1,430 *g* for 10 min. Finally, the 2.5 mL of upper layer was mixed with 2.5 mL of distilled water and 0.5 mL of ferric chloride (0.1%). After 10 min, the absorbance was measured at 700 nm against blanks that contained all reagents except the sample extracts. A higher absorbance value indicated a higher reducing power. Butylated hydroxyl-toluene (1 mg mL^-1^) was used as a positive control.

**Total phenolics. **The total phenolic contents of PJ and ZEO were determined using the Folin-Ciocalteu reagent assay according to the method of Singleton and Rossi with gallic acid as a standard.^16^ Briefly, 500 μL of PJ (1:10) and ZEO (1, 2, 4 and 8 mg mL^-1^) diluted with methanol was mixed with 2.25 mL of distilled water, and then 250 μL of Folin-Ciocalteu reagent was added. The mixture was vortexed for 1 min and was allowed to react for 5 min. Then, 2 mL of a 7.5% Na_2_CO_3_ solution was added. After incubation at room temperature for 120 min, the absorbance of each mixture was read at 760 nm. The same procedure was also applied to a standard solution of gallic acid, and a standard curve was obtained. The total phenolic contents were expressed as mg of gallic acid equivalent per g of the sample. All determinations were carried out in triplicates.

**Total anthocyanins. **The total anthocyanins were estimated by pH differential method using two buffer systems: potassium chloride buffer, pH 1.0 (25 mM) and sodium acetate buffer, pH 4.5 (0.4 M).^17^ The samples were diluted by a potassium chloride buffer until the absorbance of the sample at 510 nm wavelength were within the linear range of the Novaspec II spectrophotometer. This dilution factor was later used to dilute the sample with the sodium acetate buffer. The wavelength reading was performed after 15 min of incubation, four times per sample, diluted in the two different buffers and at two wavelengths of 510 nm and 700 nm. The total anthocyanins content was calculated as follows: 

$\mathrm{Total anthocyanis}=\frac{\left(A\times \mathrm{MW}\times \mathrm{DF}\times 100\right)}{\mathrm{MA}}$

where A = (A510 − A700) pH 1.0 − (A510 − A700) pH 4.5; MW is molecular weight (449.2); DF is the dilution factor; and MA is the molar absorptive coefficient of cyaniding-3-glucosid (i.e., 26.900). Results were expressed as mg cyaniding-3-glucoside per 100 g of juice.

**Titratable acidity, pH, total soluble solids, maturity index and total anthocyanins. **The titratable acidity (TA) was determined by titration to pH 8.1 with 0.1 M NaOH solution and expressed as g of citric acid per 100 g of juice.^18^ The pH measurements were performed using a digital pH meter (Mettler Toledo, Zurich, Switzerland) at 21 ˚C. The total soluble solids (TSS) were determined with a digital refractrometer (Erma, Tokyo, Japan). Results were reported as Brix at 21 ˚C. Maturity index was calculated by dividing total soluble solids to titratable acidity.

**Statistical analysis. **Statistical analysis of data was performed using the SPSS software package (Version 21; IBM, Armonk, USA). Analysis of variance and Tukey’s test were used to compare means of the groups.

## Results

**Chemical composition of ZEO.** Table 1 shows the compositional analysis of ZEO by GC-MS. Forty-three compounds, representing 99.37% of total oil were reported. The major components were carvacrol (59.17%), linalool (23.67%), trans-caryophyllene (3.07%) and carvacrol methyl ether (2.44%). 

**Fig. 1:**
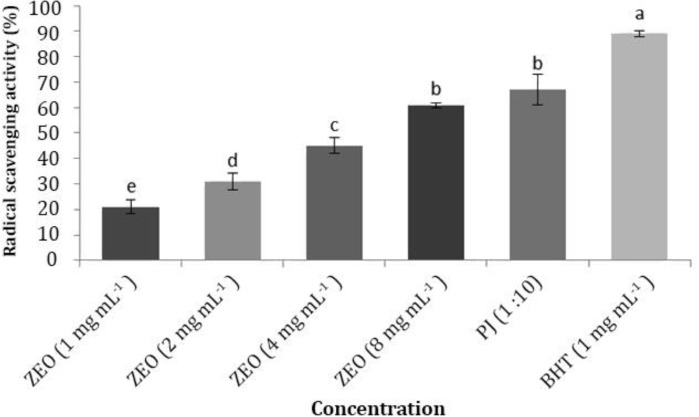
2,2-diphenyl-1-picrylhydrazyl (DPPH) scavenging activity of *Zataria multiflora *Boiss essential oil (ZEO), pomegranate juice (PJ) and butylated hydroxytoluene (BHT). Different letters indicate a statistically significant difference (*p* < 0.05).

**Antioxidative activity. **The scavenging effects of pomegranate juice (PJ), ZEO and butylated hydroxyl toluene (BHT) at different concentrations on the DPPH radical are shown in Figure 1. The DPPH was used as a free radical to evaluate antioxidant activity present in natural sources.^14^ Increasing the concentration of ZEO led to increased (*p* < 0.05) radical scavenging activity. Therefore the lowest and highest radical scavenging effect of ZEO were detected in 1 mg mL^-1^ and 8 mg mL^-1^ concentrations, respectively. The juice at 1:10 dilution level showed 67.00% radical scavenging effect. Also, ZEO at 8 mg mL^-1^ concentration exhibited similar radical scavenging activity to PJ at 1:10 dilution. Thus, ZEO showed more antioxidant ability than PJ.

**Table 1:** Chemical composition of *Zataria* *multiflora* Boiss essential oil.

**Compounds**	**KI** a	**Area (%)**
**2e-Hexenal**	858	0.06
**α-Pinene**	933	0.24
**β-Pinene**	980	0.04
**(1)Octen-3-ol**	985	0.05
**3-Octanone**	991	0.26
**3-Octanol**	1002	0.11
**α-Terpinene**	1020	0.15
**para-Cymene**	1029	0.48
**1,8Cineole**	1036	0.32
**(z-β)Ocimene**	1049	0.05
**gamma-Terpinene**	1062	0.47
**(trans)Linalool oxide**	1075	0.40
**α-Terpinolene**	1089	0.06
**cis-Linaloloxide**	1091	0.3
**Linalool**	1109	23.67
**Hotrienol**	1111	0.84
**(3-)Octanol acetate**	1122	0.13
**(1,3,8-ρ) Menthatriene**	1136	0.18
**Borneol**	1182	0.24
**Terpinene-4-ol**	1189	0.54
**α-Terpineol**	1205	1.17
**cis-Dihydrocarvone**	1211	0.19
**2,6-Dimethyl-3,5,7-octateriene-2-ol-e-e**	1219	0.16
**Thymol,methyl ether**	1236	0.30
**Carvacrol methyl ether**	1246	2.44
**Linalyl acetate**	1254	0.92
**Geraniol**	1261	0.12
**Thymol**	1293	0.06
**Carvacrol**	1304	59.17
**(delta)Elemene**	1335	0.09
**Neryl acetate**	1363	0.07
**Carvacryl acetate**	1373	0.16
**Geranyl acetate**	1383	0.11
**trans-Caryophyllene**	1426	3.07
**Aromadendrene**	1445	0.48
**α-Humulene**	1463	0.15
**(allo)Aromadendrene**	1467	0.07
**(ar)Curcumene**	1487	0.06
**Veridifloren**	1498	0.40
**Bicyclogermacrene**	1503	0.26
**β-Sesquiphellandrene**	1530	0.07
**Spathulenol**	1589	0.69
**Caryophyllene oxide**	1594	0.57
**Total**	-	99.37

a KI: Kovats indices calculated against n-alkanes on HP-5 column.

**Fig. 2:**
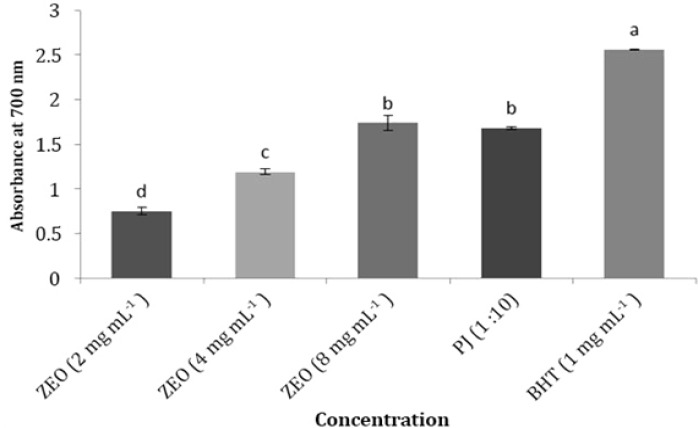
Reducing power of *Zataria multiflora *Boiss essential oil (ZEO) and pomegranate juice (PJ). Different letters indicate a statistically significant difference (*p* < 0.05).

**Reducing power. **A concentration dependent increase in reducing power was observed for ZEO (Fig. 2). As it exhibited at 2 mg mL^-1 ^and 8 mg mL^-1 ^concentrations the lowest and highest reducing power respectively. With high radical scavenging activity, ZEO at 8 mg mL^-1^ concentration exhibited similar reducing capacity to PJ at 1:10 dilution. Thus ZEO showed more reducing capacity than PJ.

**Total phenolic content. **The level of total phenolics in pomegranate juice and ZEO was 154.90 mg per 100 g and 262.52 mg per g, respectively. 

**Titratable acidity, pH, total soluble solids, maturity index and total anthocyanins. **The results of physicochemical properties of PJ were as follow: pH = 3.10 ± 0.05, total soluble solids = 16.10 ± 1.15, titratable acidity = 1.13 ± 0.14, maturity index = 14.75 ± 0.02 and total anthocyanins = 28.90 ± 3.43.

## Discussion

Results of GC-MS analytical data of compounds in ZEO were in agreement with other researchers that have also shown carvacrol to be the main component in ZEO.^19^ The quantity of these compounds can vary due to harvesting season, plant age, soil, climate, geographical sources, herb drying method and extraction method.^20^

The results of DPPH in ZEO in the present study were not in agreement with the findings of Moradi *et al.* (ZEO in 10 mg mL^-1 ^concentration with 34.00% radical scavenging effect)^13^ and Aliakbarlu *et al.* (ZEO in 2 mg mL^-1 ^concentration with 88.00% radical scavenging effect).^21^ Moreover, the findings of the 2 latter studies were not in agreement with each other. These differences may be due to changes in culture, harvest and drying conditions that lead to diversity in constituents of ZEO. The DPPH of PJ of Rabbab-e-Neyriz cultivar was the highest among other cultivars of Iranian pomegranates.^22^ That is probably due to its high amount of total anthocyanins which are major constituents of phenolic compounds of PJ.^10^

The antioxidative activity has been reported to be directly correlated with reducing power. The reducing properties are generally associated with the presence of reductones.^23^ Gordon reported that the antioxidant action of reductones is based on the breaking of the free radical chain by donating a hydrogen atom.^24^ The results of reducing power in ZEO were not in agreement with findings of Aliakbarlu *et al*. (ZEO 2 mg mL^-1^ with 1.2 absorbance).^21^ This might be due to the mentioned reasons for differences in DPPH. The results of reducing power in PJ were not similar to findings of Naveena *et al*. (200 µg PJ phenolics with 1.2 absorbance).^25^^,^^26^ This might be due to using different method or different variety of pomegranate for reducing power measurement. 

The reported levels of total phenolic content for pomegranate juice in literature were between the range of 124.50 to 207.60 mg per 100 g (Ozgen *et al*.),^27^ 208.3 to 343.6 mg per 100 g (Cam *et al.*),^28^ 14.40 to 1008.60 mg per 100 g (Tezcan *et al.*),^29^ and 23.70 to 930.40 mg per 100 g ( Mousavinejad *et al*.).^22^ These differences may be due to various varieties. In a study by Zangiabadi *et al*. the phenol content for ZEO was 322.00 ± 2.90 mg gallic acid mL^-1^.^30^ Aliakbarlu *et al*. showed that the phenol content for ZEO was 44.81 mg GAE per g of sample.^21^ These differences are related to many factors, such as geographic location, environmental and climate conditions, season of growth, soil type, storage, and processing conditions that can influence the levels of phenolics compounds. The phenol contents are important vegetable antioxidant compounds, because their hydroxyl groups have the inhibitory potential for radicals.^31^ Many researches have reported that there is a relation between the phenol content and antioxidant activity, but some researchers showed that, there may be no relation at all.^31^

The pH value in the current study was lower than those reported by Tehranifar *et al*. of other pomegranate cultivars grown in Iran.^32^ Our results indicated that the pomegranate juice (Rabbab-e-Neyriz) was the most acidic crop among other Iranian cultivars. Our result relating to the total soluble solids was higher than values observed (11 to 15 Brix) on twenty Iranian cultivars by Tehranifar*et al*.^32^ The titratable acidity content was 1.13. Similarly, Tehranifar *et al*. and Fadavi *et al*. reported same results about other Iranian cultivars.^32^^,^^33^ The maturity index (TSS/TA) is responsible for the taste and flavor of pomegranate, which some authors have used it for classifying pomegranate cultivars. This classification has been optimized for Spanish cultivars: maturity index (MI) = 5 to 7 for sour, MI = 17 to 24 for sour-sweet and MI = 31 to 98 for sweet cultivars.^34^ Regarding our result about MI (14.75), Rababe-Neyriz pomegranate can be ordered as sour-sweet. Anthocyanins are members of phenolics compounds that contribute to the red, blue, or purple color of many fruits, including pomegranate juice, and they are well-known for their antioxidant activity. Rabbab-e-neyriz cultivar is a crop with a high amount of anthocyanin (28 mg cy-3-glu per 100 g). Thus, it may be claimed that this cultivar is one of the most antioxidant crops among other Iranian cultivars. According to the results of Tehranifar *et al*. total anthocyanin values ranged between 5 to 30 mg cy-3-glu per 100 g among twenty Iranian cultivars. ‘Malase Yazdi’ had the highest amount of total anthocyanins (30.11mg cy-3-glu per 100 g) among the other cultivars and the rest were below 11mg cy-3-glu per 100 g.^32^

In the present study, physicochemical properties, chemical compositions and anti-oxidative activities of Rabbab-e-Neyriz pomegranate (*Punica granatum* L.) juice and ZEO were determined. The ZEO showed more potent anti-oxidative activity than pomegranate juice. Also its total phenolics content were higher than PJ. Moreover, ZEO exhibits its food preservative effects in low amounts. Its usage in food is limited due to the vigorous taste and aroma,so it cannot be directly used in high amounts as food preservative. However, Rabbab-e-Neyriz pomegranate (*Punica granatum* L.) juice and its products can be directly used in various foods due to their wonderful taste and high palatability. It can also increase food shelf life by its high antioxidant activities. Therefore ZEO maybe can be used in low amounts as a portion of coating of dipped foods in PJ and its products in order to remain and prolong its effects on foods. 
